# “Wife, widow, or warrior?”: Media representation of women politicians in Southern Africa

**DOI:** 10.3389/fsoc.2026.1838215

**Published:** 2026-09-23

**Authors:** Tigere Paidamoyo Muringa

**Affiliations:** Journalism Programme, Department of Media, Communication and Language, Faculty of Arts and Design, Durban University of Technology, Durban, South Africa

**Keywords:** feminism, gender tropes, media representation, Southern Africa, women leaders

## Abstract

A growing body of literature recognises the media’s role in shaping political identities and leadership legitimacy. Studies suggest that women presidential candidates in post-colonial Southern Africa are often framed through historical, cultural, and gendered archetypes that limit their political agency. Drawing on feminist media theory, framing theory, and Critical Language Awareness (CLA), this study analyses how media language constructs women presidential aspirants as political wives, widows continuing male legacies, or warriors fighting the system. The study employs content analysis of 122 news articles published by three Southern African online outlets: NewsDay in Zimbabwe, IOL in South Africa, and Malawi24 in Malawi. The findings show that media narratives restrict women’s political agency by compelling them to conform to predetermined roles rather than recognising them as independent presidential contenders. While some coverage suggests that women can transcend these archetypes, dominant narratives continue to reproduce traditional gender roles in politics. From a CLA perspective, these findings demonstrate that media language does not merely describe women’s political participation; it actively shapes public understanding of their legitimacy, competence, and authority. The study argues for greater media accountability and a CLA-informed reporting framework that promotes equitable, diverse, and gender-sensitive representations of women in political leadership.

## 1 Introduction

In democratic societies, the media is expected to function as a watchdog by facilitating balanced political debate and providing equal space and equitable representation for all candidates, irrespective of gender (Coffie and Medie, 2021). Media coverage should provide women political leaders with visibility that recognises their legitimacy as contenders for the highest office, contributing to inclusive democratic participation and the advancement of gender equity (Asare and Agomor, 2023). In this process, news outlets and digital platforms should act as critical sites for dismantling entrenched patriarchal stereotypes rather than reinforcing them (Nkoa et al., 2023). From a Critical Language Awareness (CLA) perspective, however, the media’s democratic role depends not only on whose voices receive coverage, but also on how linguistic choices and frames construct political legitimacy. Media language can influence people’s attitudes and behaviour and significantly affect public confidence in democracy, politics, and the rule of law (Darics et al., 2025). CLA is therefore important for examining how apparently routine words, labels, metaphors, and narrative structures may reproduce unequal social and political power relations. The role of the media is particularly critical in contexts where elections are both a mechanism of democratic renewal and a contested terrain for gendered power (Sazali and Basit, 2020).

Across much of Southern Africa, the media continues to perpetuate narrow frames that constrain women leaders, portraying them less as autonomous contenders and more through cultural archetypes that cast them as wives, widows, or exceptional “warriors” (Muringa et al., 2024). These reductive representations stem from deep-rooted cultural and political histories in which political authority has long been associated with male leadership. Women seeking the presidency are frequently depicted in ways that diminish their independent agency, such as Nkosazana Dlamini-Zuma as the warrior confronting a male-dominated political system (Muringa and McCracken, 2021). Such archetypes not only limit women’s visibility but also reinforce patriarchal discourses that shape the electorate’s perceptions of leadership and suitability for office (Cornell et al., 2021). Viewed through CLA, these archetypes are not neutral descriptions. They represent ideological language choices that can naturalise gendered political hierarchies and make the exclusion of women from executive authority appear reasonable or inevitable. The broader problem, therefore, is that while women in Southern Africa have entered presidential races, their campaigns are consistently undermined by media frames that delegitimise their authority and perpetuate structural exclusion from political power (Vyas-Doorgapersad and Shava, 2022).

Recent scholarship highlights the multifaceted challenges faced by women presidential aspirants in the region (Gouws, 2023). Muringa et al. (2024) demonstrate that media in South Africa construct narratives asserting that society is “not ready for a ‘she’ president,” thereby normalising doubt around female leadership. Tembo (2024) further illustrates how Malawian women encounter gendered political violence on social media, where fear and intimidation silence their participation. Similarly, Muringa and McCracken (2021) found that coverage of Nkosazana Dlamini-Zuma during the ANC’s 54th National Conference emphasised her gender and personal relationships, privileging her male opponents. In Zimbabwe, Ncube and Yemurai (2020) reveal that women candidates faced targeted abuse on Twitter during the 2018 elections, reinforcing a climate of discrimination. Cornell et al. (2021) demonstrate how South African media discourses of political violence often cast women as vulnerable victims rather than autonomous leaders. Likewise, Matsilele and Nkoala (2023) note that while women politicians adopt digital strategies to reframe their public image, their messaging is still constrained by entrenched partisan and gendered biases. Collectively, these studies confirm that while women contest the presidency, their legitimacy continues to be filtered through limiting archetypes (Chikafa-Chipiro, 2023).

Despite attempts in previous studies, existing literature has focused on single-country case studies, with little attention given to comparative analysis of women presidential candidates in Southern Africa. Surprisingly, the specific archetypes of wife, widow, and warrior have not been systematically examined as a framework for understanding how media across different countries construct female presidential ambition (Sandwell, 2022). Existing research has also paid insufficient attention to the CLA dimensions of these representations, particularly how specific lexical choices, evaluative descriptions, presuppositions, and recurring frames encode gendered assumptions about political authority. This gap leaves unclear the extent to which such frames reinforce patriarchal discourses across borders or whether women can transcend them to redefine political leadership (Mechkova and Carlitz, 2020).

Drawing on Feminist Media Theory, Framing Analysis, and Critical Language Awareness, this study examines media coverage of three prominent women political candidates: Grace Mugabe in Zimbabwe, Joyce Banda in Malawi, and Nkosazana Dlamini-Zuma in South Africa (Illés and Mwangi, 2024). By systematically analysing how news media frames these candidates, the study identifies the dominant archetypes at play, evaluates how these archetypes restrict or enable women’s political agency, and explores whether alternative frames of leadership emerge (Gakahu, 2024). The CLA perspective extends this analysis by examining how language choices construct, legitimise, or challenge gendered relations of power. The study further positions CLA as a future-oriented competence through which journalists, editors, and media audiences can recognise and contest discriminatory political discourse rather than reproduce it. Three key questions guide this inquiry:

- In what ways do media representations in Southern Africa construct and frame women politicians through historical and cultural archetypes?
- How do media portrayals of women politicians reflect and reproduce patriarchal discourses in African societies?
- How can a CLA-informed reporting framework be developed to guide more equitable and gender-sensitive portrayals of women politicians in Southern African media?

After this introduction, the paper is structured as follows. The next section discusses the political systems in Malawi, Zimbabwe and South Africa. The following section then outlines the theoretical framework and literature guiding the study, followed by a description of the methodology. The subsequent sections present findings on the coverage of Grace Mugabe, Joyce Banda, and Nkosazana Dlamini-Zuma, and a comparative discussion of the archetypes. The paper concludes with reflections on the implications of these findings for media accountability, critical language awareness, and the promotion of gender-inclusive political representation.

## 2 Political systems in Malawi, Zimbabwe and South Africa

The political structures of South Africa, Zimbabwe, and Malawi are characterised by distinctive cultural and patriarchal influences, which shape women’s involvement in politics, with varying success in closing the gap between constitutional and substantive representation. South Africa boasts the highest female representation, with approximately 43–46% of parliamentarians being women through proportional representation and the ruling ANC’s voluntary zebra system, which alternates male and female candidates on the party list (Kgobe and Ramoroka, 2024). Nevertheless, cultural patriarchy continues to prevent women from occupying top political roles. Women remain excluded from powerful political positions, with no female head of state elected and limited representation within influential party structures, reflecting the enduring impact of homosocial capital (Twalo, 2024). The ongoing challenges in achieving substantive gender equality, despite numerical gains, highlight a tension between feminist aspirations and cultural barriers that continue to inhibit women from reaching top decision-making positions.

Zimbabwe, despite having a constitutional provision on gender equality through the hybrid electoral system, still bears a deeply rooted patriarchal culture in its politics (Ringson, 2025). The hybrid system, intended to empower women, has in effect disempowered them and relegated them to secondary seats in parliament, as ruling parties often use the provision as an excuse not to give women a fair chance in competitive elections (Mtero, 2025). Patriarchal culture, electoral violence, and the exclusion of women from the core centres of politics have all contributed to women’s disempowerment and made it difficult for them to participate actively in the country’s political life (Nare, 2022). Consequently, gender politics in Zimbabwe are influenced by both institutional and cultural factors, and the constitutional provision has fallen short in challenging the existing political norms (Ndhlovu, 2024).

Malawi, which has the lowest rate of women’s participation in parliament at only 21%, faces an even more challenging situation, with issues stemming from the FPTP system and the entrenched patriarchal culture within its politics (Ludaka and Cissé, 2025). The costs and competitiveness of the system, coupled with weaker provisions in voluntary systems, hinder women’s chances of achieving significant political breakthroughs (Lihiru, 2024). Patriarchal culture is deeply embedded in Malawi’s political landscape, where politics remains largely regarded as a male domain, and women have considerably lower chances of participating or reaching influential positions within the system (Ludaka and Cissé, 2025). The absence of a strong, legislated quota system further complicates matters, as the issue is heavily affected by the complex phenomena of patriarchy and feminism.

Although Malawi, Zimbabwe, and South Africa are geographically proximate and share certain colonial and post-colonial legacies, their political systems and societies are not interchangeable, and the patterns of women’s exclusion described above should not be read as evidence of a single, undifferentiated “Southern African” experience. Each country’s gendered political landscape is also embedded within, and should be read alongside, broader patterns of women’s participation in the public sphere beyond formal electoral politics. In South Africa, women’s relatively high numerical representation in parliament coexists with persistent gender gaps in senior corporate leadership, the judiciary, and traditional governance structures, and with comparatively high rates of gender-based violence that continue to constrain women’s freedom of movement and public visibility (Twalo, 2024). In Zimbabwe, women’s participation in public life is shaped by a dual legal system in which customary law continues to govern significant aspects of family, land, and inheritance rights in ways that limit women’s economic independence and, by extension, their capacity to mount sustained political campaigns (Nare, 2022; Ndhlovu, 2024). In Malawi, where women’s labour-force participation in agriculture is high but largely informal and undervalued, entrenched customary authority continues to regulate women’s roles in community decision-making, reinforcing a perception of politics as an extension of male public authority rather than a space women can occupy on equal terms (Ludaka and Cissé, 2025). These patterns suggest that women’s exclusion from formal political leadership in each country is connected to, rather than separate from, broader constraints on women’s voice and visibility in the public sphere more generally. This broader connection is treated as part of the interpretive context for the media analysis that follows, even though the empirical focus of the present study remains confined to media coverage of the three presidential aspirants discussed below.

### 2.1 The presidential aspirants

Grace Mugabe, Joyce Banda, and Nkosazana Dlamini-Zuma are three prominent political figures in Southern Africa whose political careers have been deeply intertwined with their relationships to male political figures and whose media portrayals have been shaped by gendered narratives (Mangena, 2022; Muringa and McCracken, 2021). Understanding the political contexts of their careers is crucial to interpreting their media representation and the implications for women’s political agency in Africa (Mangena et al., 2021).

Grace Mugabe, the wife of former Zimbabwean President Robert Mugabe, became a prominent political figure during her husband’s rule (Mangena, 2022). Although she was not a presidential candidate in Zimbabwe’s national elections, her potential candidacy for the presidency became a focal point in Zimbabwean politics in the mid-2010s (Mangena, 2022). In 2014, Grace Mugabe was appointed the head of the ruling Zimbabwe African National Union—Patriotic Front (ZANU-PF) Women’s League and was considered by many to be a possible successor to her husband (Mangena, 2022). She gained prominence through her role in ZANU-PF and was involved in the political dynamics that led to the November 2017 military coup, which ultimately ousted Robert Mugabe from power (Mangena et al., 2021). The framing of Grace Mugabe’s political career often reflected her association with her husband’s legacy, and she was frequently presented as a political heir rather than an independent political figure (Mangena, 2022). While Grace Mugabe’s leadership was often framed in terms of “divine sanction,” it is important to note that Zimbabwe is a country with a contentious democratic history, marked by political repression and human rights violations (Mangena et al., 2021). This context is key in understanding the framing of her leadership (Mangena et al., 2021). Grace Mugabe’s portrayal as a “divinely sanctioned” leader reflects Zimbabwe’s long-standing political culture, which was largely dominated by the Mugabe regime and marked by electoral fraud and the suppression of opposition parties. Thus, her leadership was not seen as earned through democratic processes but rather as a continuation of the autocratic leadership model that had defined Zimbabwean politics under her husband’s rule (Mangena et al., 2021).

Joyce Banda was Malawi’s President from 2012 to 2014 after her predecessor, Bingu wa Mutharika, died (Kondowe et al., 2014). A DPP member, she was Vice President before her presidency, which she assumed under Malawi’s constitution upon Mutharika’s death (Kondowe et al., 2014). Her leadership continued her late husband’s legacy and included economic and political reforms, such as liberalising Malawi’s economy and engaging with international donors (Kondowe et al., 2014). Her presidency was notable in women’s political legitimacy and empowerment, stepping into a traditionally male-dominated leadership role (Kondowe et al., 2014). Like Grace Mugabe, her leadership was often viewed through family and political inheritance lenses, highlighting how female political legitimacy remains linked to male authority in Malawi and beyond (Kondowe et al., 2014).

Nkosazana Dlamini-Zuma, a former South African Minister of Home Affairs and Chairperson of the African Union Commission, ran for ANC president in 2017 (Muringa and McCracken, 2021). Unlike the other two women in this review, she aimed to lead the ANC, positioning herself to challenge for the presidency in 2019 (Muringa, 2019). Her candidacy was symbolic, representing a potential shift from the ANC’s male-dominated leadership (Muringa and McCracken, 2021). Her political career is often framed by her achievements and familial ties, as the ex-wife of former President Jacob Zuma (Adjin-Tettey and Muringa, 2026). The media portrayed her campaign as a challenge to male dominance, sparking debate about women’s roles in the ANC (Muringa, 2019). She highlighted her domestic and continental political record as qualifications to lead the ANC and ultimately South Africa (Muringa and McCracken, 2021). However, the media often depicted her as an outsider challenging the male establishment, reinforcing the warrior archetype that casts women leaders as exceptional and requiring proof of legitimacy beyond that of male leaders (Muringa, 2019, 2026).

## 3 Theoretical framework and literature

### 3.1 Feminist media theory

Feminist media theory provides the foundation for illuminating how news discourse and digital media uphold patriarchal structures, portray women through stereotypes, and establish strict boundaries for political legitimacy around them (Muringa et al., 2024). This theory is particularly relevant to the prototype of wife, widow, and warrior because it explains why female leaders are often framed within narratives linked to domesticity, male affiliation, or heroic struggle, rather than leadership itself. Through employing feminist media theory, this study explores how media discourses shape, confine, and occasionally subvert the political personas of Grace Mugabe, Joyce Banda, and Nkosazana Dlamini-Zuma.

Feminist media theory emerged as a response to the exclusion and misrepresentation of female voices in communication research (Steiner, 2014). Early feminist critiques highlighted that the media often failed to portray women as credible actors, instead relegating them to private or symbolic roles (Ceulemans and Fauconnıer, 1979). The core of the theory is rooted in the idea of representation as a cultural process that creates and disseminates meanings surrounding gender and power (Sazali and Basit, 2020). In political communication, such representations are neither accidental nor neutral; they shape public perceptions of leadership and authority. African studies support that female politicians are frequently depicted in relation to men or through traits associated with femininity, such as nurturing or moral purity (Coffie and Medie, 2021; Asare and Agomor, 2023). These assumptions underscore the importance of feminist media theory as a vital tool for analysing the gendered logics within presidential news.

Recent research highlights both the strengths and challenges of applying feminist media theory to African contexts. For instance, Muringa and McCracken (2021) demonstrate how South African media portrayed Dlamini-Zuma in relation to Jacob Zuma, highlighting the persistence of patriarchal logics. Tembo (2024) illustrates how Malawian women experience political violence on social media, where fear discourses are used to suppress their participation. Similarly, Ncube and Yemurai (2020) report that Zimbabwean women aspirants faced targeted opprobrium on Twitter, reinforcing their exclusion from politics. Cornell et al. (2021) also reveal that discourses of violence in South Africa often depict women as passive victims and political leaders. Meanwhile, some researchers acknowledge women’s agency in managing hostile media spaces. Matsilele and Nkoala (2023) demonstrate that female politicians utilise digital tactics, such as metavoicing, to counter partisan attacks, while Chikafa-Chipiro (2023) shows how Zimbabweans use social media platforms to challenge exclusion. These debates suggest that while feminist media theory evidences the ongoing patriarchy in media systems, it must also recognise women’s resistance and the fluidity of meaning.

This research employs feminist media theory to analyse how the media portray Grace Mugabe, Joyce Banda, and Nkosazana Dlamini-Zuma. It reveals that the media often frames women in gendered roles that reinforce patriarchal norms. Using feminist media theory, the study shows how such framing limits women’s agency while also highlighting instances of counter or resistant portrayals. This approach directly addresses the research questions: how women presidential candidates are represented and whether these frames reinforce or challenge patriarchal discourses.

Feminist media theory provides a strong perspective, but its application has limitations. Critics warn against assuming all women’s experiences are identical, as media representations are influenced by race, class, and local culture (Nartey, 2023). In Southern Africa, these factors mean that feminist analysis should be grounded in the region’s histories of colonialism, liberation struggles, and postcolonial development. Despite these challenges, the theory remains essential for revealing the media’s role in reinforcing gender roles and enabling researchers to critically assess how women leaders are either marginalised or supported. Concretely, this colonial and post-colonial grounding shapes the analysis in country-specific ways rather than treating Southern Africa as a single, homogeneous discursive context. In Zimbabwe, Grace Mugabe’s portrayal cannot be separated from a political culture in which legitimacy has historically been constructed through proximity to liberation-war credentials and the ruling party’s leadership rather than through competitive electoral mandate; her framing as a dynastic heir is therefore read as an extension of this broader post-colonial pattern of personalised, legacy-based authority, not solely as an instance of gendered media bias in the abstract. In South Africa, Nkosazana Dlamini-Zuma’s coverage is read against the African National Congress’s status as a former liberation movement, in which leadership legitimacy continues to be substantially anchored in struggle credentials and party seniority, a context that shapes the salience of the “liberation motherhood” frame discussed later in this study. In Malawi, Joyce Banda’s ascent through constitutional succession rather than electoral mandate is read against a political system with comparatively weak quota provisions and a still-emerging tradition of women’s competitive candidacy for the highest office, which this analysis treats as a condition for, rather than merely a backdrop to, her “caretaker” framing. These country-specific trajectories are revisited throughout the Findings and Discussion below.

This research is grounded in feminist media theory because it explains why female presidential candidates are often portrayed using simple archetypes and how these images reinforce patriarchal norms. It offers a conceptual framework for analysing both types of representation and their wider implications for feminist political agency in Southern Africa. By applying the theory to the cases of Mugabe, Banda, and Dlamini-Zuma, the study contributes to the development of media, gender, and power theory and offers a comparative view of feminist media analysis across Africa.

### 3.2 Framing theory

Framing theory is the main tool used to analyse how the media constructs and sustains dominant narratives about women presidential candidates in Southern Africa. Entman (1993) describes framing as the selection of specific aspects of reality and their highlighting in communication to influence problem definitions, causal explanations, moral judgements, or proposed solutions. Entman has since revisited and extended this original model. Entman and Usher (2018) argue that, in increasingly fragmented and digitally mediated public spheres, the cascading flow of frames from political and media elites to audiences is no longer monopolised by a small number of gatekeepers. Instead, frames are contested by networked publics that can amplify, ignore, or recast elite narratives through social sharing and algorithmic curation. This refinement is directly relevant to the present study because the corpus is drawn from online news platforms whose framing choices are shaped not only by editorial selection, in Entman’s original sense, but also by digital virality and audience engagement metrics that the 1993 model did not anticipate.

Framing also provides an important connection between the study and Critical Language Awareness (CLA). Within CLA, framing forms part of metalinguistic awareness because it requires readers, journalists, and researchers to reflect critically on how language is selected, organised, and presented to construct particular versions of political reality. Metalinguistic awareness extends analysis beyond what a news report communicates to examine how lexical choices, labels, metaphors, grammatical structures, evaluative descriptions, presuppositions, and patterns of emphasis or omission influence interpretation. Framing analysis therefore operationalises this dimension of CLA by making visible the linguistic and rhetorical choices through which media discourse legitimises certain political identities while marginalising others.

Research shows that media frames actively shape public perceptions of leaders and function as influential agents rather than merely reflecting political events (Coffie and Medie, 2021; Muringa and McCracken, 2021). This study employs framing analysis to examine how outlets such as IOL, NewsDay, and Malawi24 highlighted or downplayed the political legitimacy of Joyce Banda, Nkosazana Dlamini-Zuma, and Grace Mugabe. The analysis uncovers archetypes such as wife, widow, and warrior in headlines and news stories, demonstrating how these portrayals reinforce societal expectations about women’s roles in leadership (Sazali and Basit, 2020; Tembo, 2024). From a CLA perspective, identifying these archetypes involves recognising that such labels are not merely descriptive. They are metalinguistic and ideological resources that influence how audiences understand women’s political competence, independence, and entitlement to authority.

This study highlights the strength of framing theory in revealing the ideological foundations of media discourse. Previous research indicates that framing serves as a potent tool for maintaining patriarchal narratives by depicting women’s leadership as unusual, dependent, or politically less legitimate (Cornell et al., 2021; Asare and Agomor, 2023). For example, framing Banda as a widow carrying on her husband’s legacy or Dlamini-Zuma as a fierce warrior defines their political identities and restricts their recognition as ordinary and legitimate political contenders. A CLA-informed framing analysis interrogates the language through which these identities are produced and encourages critical reflection on the assumptions that such representations make appear normal or self-evident. Thus, this research employs framing to explore how cultural archetypes are strategically utilised across Zimbabwe, Malawi, and South Africa to shape perceptions of women’s agency in presidential politics.

Framing theory consequently offers a conceptual connection between micro-level textual choices and macro-level patriarchal ideologies, showing how frames operate both as storytelling devices and as mechanisms of political exclusion (Chikafa-Chipiro, 2023; Ncube and Yemurai, 2020). Its integration with CLA further positions framing analysis as a future-oriented competence. It equips media practitioners and audiences to recognise, question, and reconstruct gendered representations rather than passively accepting the ideological meanings embedded in political reporting.

### 3.3 Archetypes in media representations of female political leaders

The media representation of women in political leadership is shaped by deeply ingrained gendered archetypes, such as *wives*, *widows*, and *warriors* (Laher, 2014). These archetypes play a crucial role in shaping how women’s political legitimacy is framed in both African and global contexts. As studies in both regions have demonstrated, these portrayals often position women either as extensions of male figures or as exceptional outliers who must deviate from political norms to gain recognition (Muringa and McCracken, 2021). The *wife* archetype, for example, often reduces women to mere extensions of male political power, while the *widow* archetype can either empower or further subjugate women depending on its framing. Similarly, the *warrior* archetype, while ostensibly empowering, often isolates women as extraordinary political figures rather than as ordinary leaders. These archetypes not only constrain women’s political agency but also reinforce the patriarchal structures that continue to dominate global political spheres. This literature review synthesises both African and international sources to explore how these archetypes operate and shape public perceptions of women politicians, contributing to the broader conversation on gendered media representations.

### 3.4 The wife archetype: political legitimacy through relationships to male figures

The *wife* archetype in media framing of female politicians consistently links women’s political agency to their relationships with male political figures, thereby limiting the full recognition of their political legitimacy (Muringa and Ndlovu, 2026). Women in political power are often depicted as extensions of their male counterparts, either as wives or political heirs (Chernenko, 2025). In Southern Africa, figures such as Grace Mugabe and Nkosazana Dlamini-Zuma are frequently framed through this lens, with their political legitimacy tied to their husbands’ legacies (Muringa and McCracken, 2021; Mangena, 2022). These portrayals undermine the individual achievements of these women by framing them as secondary to male authority. This narrative of political legitimacy through relationship to male figures is not limited to Africa but is a global phenomenon. In the United States, Hillary Clinton’s political career was often portrayed in terms of her relationship to her husband, Bill Clinton, and similarly, Sonia Gandhi’s political rise in India was framed as a continuation of her husband’s legacy. As Ceulemans and Fauconnıer (1979) highlight, the media often obscures the autonomy of women politicians by focusing on their relationships to male figures, whether through marriage or political inheritance. This framing perpetuates a patriarchal understanding of political leadership, suggesting that women are legitimate leaders only when they are connected to powerful men, thereby reinforcing gendered barriers to women’s political agency (Sazali and Basit, 2020).

In this context, the *wife* archetype is not just a symbolic role but a deeply limiting construct that defines women’s political participation as secondary to the male-dominated political systems they enter. Such framing minimises the significance of women’s own contributions, thus depriving them of full political recognition. As studies in Southern Africa, India, and the US show, the *wife* archetype continues to influence how women’s political careers are perceived, constraining their political legitimacy by positioning them as extensions of male power rather than as leaders in their own right (Ncube and Yemurai, 2020; Shin, 2022).

### 3.5 The widow archetype: political entry through male loss

The *widow* archetype provides women with an entry into political life, but this opportunity often comes with the cost of their political legitimacy being framed as derivative rather than independent (Ellison, 2025). The ascension of Joyce Banda to the presidency in Malawi is frequently depicted as a continuation of her late husband’s legacy, positioning her political authority as a product of her widowhood rather than her own merits (Tembo, 2024). This framing of widowhood as a means of gaining political legitimacy echoes broader global patterns where political leaders like Sonia Gandhi in India and Benazir Bhutto in Pakistan were seen as heirs to political dynasties (Shin, 2022). The media, in both the Global South and Western contexts, often depicts widowhood as a politically sanctioned avenue for women’s leadership, framing their ascension to power as a form of political inheritance.

However, this framing simultaneously reinforces the notion that women’s political legitimacy is tied to their relationship to male figures. As observed by Ncube and Yemurai (2020), female politicians in Zimbabwe often face harassment that underscores their subordination to male political figures, whether through marriage or widowhood. Similarly, the portrayal of Banda and other female political leaders in posthumous contexts highlights how widowhood serves as a site of both empowerment and subordination. While widowhood can provide women with political opportunities, it simultaneously limits their political agency by framing their leadership as a continuation of male authority (Tembo, 2024; Ncube and Yemurai, 2020). These dynamics are not exclusive to Africa; they reflect global trends where women’s leadership is often tied to their relationship with male figures, reinforcing the patriarchal notion that women can only lead in the wake of male figures (Shin, 2022).

### 3.6 The warrior archetype: exceptionalism as empowerment and exclusion

The *warrior* archetype positions women as exceptional figures who must perform above and beyond the expected norms of political leadership to gain legitimacy. This archetype is frequently applied to women who challenge male dominance in political spaces, such as Nkosazana Dlamini-Zuma during the ANC’s 54th National Conference (Muringa and McCracken, 2021). While the *warrior* archetype may seem empowering, it simultaneously reinforces exclusion by framing women as exceptions rather than as ordinary political leaders. This exceptionalism, as argued by Nartey (2023), often forces women to overperform symbolically to gain political credibility, an expectation that is not typically placed on male political figures. This framing is evident globally, where female leaders, such as Margaret Thatcher in the UK and Indira Gandhi in India, were often depicted as exceptional figures who had to transcend traditional gender norms to be taken seriously in the political sphere (Nartey, 2023).

The warrior framing works to both empower and marginalize women. While it allows women to gain visibility in male-dominated spaces, it also positions them as outsiders to the political norm. As Mavin and Yusupova (2024) suggest, the portrayal of women as *warriors* further marginalises them by emphasising their deviation from the political norm, thus reinforcing the idea that leadership is inherently masculine. This creates a paradox in which women are both celebrated for their political strength and simultaneously excluded from regular political leadership roles due to the exceptionalist framing that defines their legitimacy (Mavin and Yusupova, 2024; Nartey, 2023).

### 3.7 Cultural and historical continuities in archetypal representations

The persistence of the *wife*, *widow*, and *warrior* archetypes in media representations of female politicians is not a contemporary phenomenon but is rooted in cultural and historical narratives (Agu, 2024). In Southern Africa, Kalichini (2023) illustrates how women’s roles in the liberation struggles were often framed as supportive rather than independent, reinforcing the idea that women’s political agency is secondary to male leadership. Similarly, studies on women’s representation in European and Yugoslav resistance movements highlight a pattern of exceptionalism, in which women were portrayed as symbols of sacrifice or support rather than as active political agents (Jelušić, 2022). These historical continuities suggest that the media framing of women politicians is deeply influenced by colonial and post-colonial gendered expectations that limit women’s political visibility.

Globally, these patterns are observed across various cultural contexts, from the framing of women in resistance movements in Europe to the portrayal of female political leaders in India and the US (Shin, 2022; Jackson, 2022). Comparable dynamics have also been documented beyond these regions: in Latin America, prominent women presidents such as Cristina Fernández de Kirchner and Dilma Rousseff have likewise been framed in relation to family political legacies, although with somewhat greater latitude to assert independent authority once in office (Agu, 2024), while in the Middle East and North Africa, women in public life are frequently depicted primarily as wives or daughters of male leaders, a framing that further restricts their independent political agency (Omar, 2025). These additional regional comparators are introduced here, and revisited in the Discussion, to situate the Southern African cases within a wider, cross-regional pattern of gendered media framing rather than treating them as a uniquely African phenomenon. In each case, women are either depicted as *wives* and *widows*, or as *warriors*, reinforcing the notion that their political agency is always tied to male figures or exceptional acts of defiance against gendered norms. These archetypes have thus shaped not only the political trajectories of women in the Global South but also the global narratives of women’s roles in politics.

The media’s framing of female politicians through the archetypes of *wives*, *widows*, and *warriors* reveals the deep-rooted gendered assumptions that shape women’s political legitimacy (Agu, 2024). These archetypes operate as mechanisms of exclusion, positioning women as either extensions of male power or as exceptional figures who must overperform to gain recognition. The global scholarship reviewed in this study shows that these archetypes are not unique to Southern Africa but reflect broader, cross-cultural patterns of gendered media representation. While these archetypes provide women with a platform to enter politics, they simultaneously constrain their political agency by framing their leadership as either dependent on male figures or as extraordinary exceptions to political norms.

## 4 Methodology

This study employed qualitative content analysis to examine the media portrayals of Southern African women presidential aspirants. The focus was on how the media framed these women through cultural and historical archetypes, *wives*, *widows*, and *warriors*, that either reinforced or challenged their political legitimacy. A qualitative content analysis is an effective method in mass communication research, as it allows for systematic examination of text to uncover underlying meanings and patterns in media representations (Coffie and Medie, 2021; Muringa and McCracken, 2021). This approach not only facilitates an in-depth understanding of gendered portrayals but also highlights the patriarchal discourses that shape political legitimacy for female leaders.

Although the study is framed as a qualitative content analysis, its design draws as much on discourse-analytic and framing-analytic traditions as on conventional, frequency-based content analysis. Conventional content analysis typically requires a sample large enough to support claims about the prevalence or representativeness of particular categories; the present study instead pursues an interpretive, case-oriented reading of a purposively selected corpus, in which the depth and texture of textual analysis, rather than statistical generalisability, grounds the claims advanced. The final sample of 122 articles, unevenly distributed across the three outlets (26 from *NewsDay*, 20 from *IOL*, and 76 from *Malawi24*), is accordingly treated as sufficient to illustrate recurring discursive patterns within each case rather than as a representative sample from which prevalence claims about Southern African media in general could be drawn. This positions the study closer to a qualitative, framing-informed discourse analysis than to quantitative content analysis; the term “content analysis” is retained here to describe the systematic, code-based reading of the corpus described below, not to claim statistical generalisability.

### 4.1 Case study selection and time span

Three prominent online news outlets were chosen for this study: *NewsDay* (Zimbabwe), *IOL* (South Africa), and *Malawi24* (Malawi). These outlets were selected based on their significant readership, influence on national political discourse, and regular coverage of presidential candidates in Southern Africa. Each candidate’s media coverage was analysed across different time spans to account for the varying lengths of their political campaigns and leadership roles. The chosen periods for data collection were justified as follows:

- *Nkosazana Dlamini-Zuma*: Coverage from 1 October to 31 December 2024, during her campaign for the African National Congress (ANC) presidency. The period of her candidacy within the ANC was selected to capture media portrayals of her political aspirations and positioning within the party leadership race.
- *Joyce Banda*: Articles from January 2014 to September 2015, covering her presidency and political activities following her ascent to the Malawian presidency. This period is significant as it includes both her presidential campaign and her time in office, allowing for a comprehensive analysis of her leadership.
- *Grace Mugabe*: Coverage from 2016 to 2017, during her time as the president of the ZANU-PF Women’s League and her positioning as a potential successor to Robert Mugabe. This period was chosen to examine how media outlets framed her political potential and her public image as the “first lady” and political heir.

The selection of different time spans was essential for capturing the evolving media portrayals of these women at different stages of their political careers. The varying time periods also allowed for a nuanced understanding of how their roles were framed during election cycles and periods of political activity, offering a comparative lens for analysis.

A central methodological question is why these three countries, rather than other Southern African states, were selected for comparison. Although Malawi, Zimbabwe, and South Africa are geographically proximate, they are not interchangeable cases: as the preceding section on political systems shows, each has a distinct colonial trajectory, electoral architecture, and rate of women’s parliamentary representation, ranging from South Africa’s 43–46% to Malawi’s 21%. They were nevertheless selected because each is among the small number of Southern African countries in which a woman has either held the presidency or the leadership of the dominant ruling party, or has mounted a high-profile bid to do so, offering a rare opportunity to examine how media discourse responds to women’s presidential ambition within, rather than merely alongside, mainstream national politics. The three countries further represent contrasting institutional arrangements for translating gender equality commitments into political office, South Africa’s proportional-representation system with a voluntary party quota, Zimbabwe’s hybrid system with reserved seats, and Malawi’s first-past-the-post system with no legislated quota, which allows the analysis to consider how differing institutional contexts intersect with similar discursive archetypes.

It is also important to clarify the basis on which the three cases are compared. Because Grace Mugabe, Joyce Banda, and Nkosazana Dlamini-Zuma were covered by different outlets, in different decades, and within different national media systems, this study does not treat the three corpora as a single, time-controlled sample, nor does it claim that the volume or character of coverage is equivalent across cases. Rather, the comparative ambition operates at the level of the archetype: the analysis asks whether recurring gendered discursive patterns, the wife, the widow, and the warrior, are visible across cases that differ in time, place, and outlet, rather than asserting that the women’s campaigns or media environments were themselves directly comparable. This case-centred, archetype-focused logic, rather than a strictly synchronic or outlet-matched design, is consistent with the qualitative, interpretive aims of the study and is revisited in the Data Analysis section below.

### 4.2 Data collection

The data collection process involved retrieving articles from the online archives of the three selected news outlets. Using search terms such as “Nkosazana Dlamini-Zuma ANC presidency,” “Joyce Banda Malawi elections,” and “Grace Mugabe successor,” the research team identified relevant articles. Broader search terms like “women president,” “female leadership,” and “first lady politics” were also used to ensure comprehensive coverage of each candidate’s portrayal across different political contexts. The articles were then reviewed based on inclusion criteria: only those that addressed the candidate’s political role, leadership ambitions, or public image were included in the analysis. Articles that focused solely on personal life or non-political events were excluded.

A final sample of 122 articles was retained for analysis: 26 from *NewsDay* (Zimbabwe), 20 from *IOL* (South Africa), and 76 from *Malawi24* (Malawi). This sample was deemed sufficient to capture the diverse ways these women were portrayed across different media outlets and political environments.

### 4.3 Data analysis

The data analysis employed Braun and Clarke’s (2006) thematic analysis methodology, which is particularly useful for identifying, analysing, and reporting patterns within qualitative data. The analysis process was iterative, involving repeated cycles of data coding and theme development to ensure a comprehensive understanding of each candidate’s media framing. Initially, the researcher familiarised themselves with the collected data by reading the articles and identifying recurring discourses related to the *wife*, *widow*, and *warrior* archetypes. These initial codes focused on identifying how the media constructed each candidate’s political legitimacy, leadership qualities, and public perception.

It is important to clarify the analytic logic underpinning the wife, widow, and warrior typology, since this bears on whether the study is inductive or deductive. The three archetypes were not generated inductively from the corpus alone: they were derived deductively from existing feminist media scholarship on gendered political leadership (Sazali and Basit, 2020; Muringa and McCracken, 2021) and used as theory-informed sensitising concepts to structure the initial coding frame. Within each archetype, however, the specific codes, subcategories, and illustrative articles reported in Table 1 were developed inductively, through repeated, close readings of the corpus, rather than fixed in advance of data engagement. The analytic process is therefore best described as abductive, moving iteratively between theory and data: a theory-driven typology supplied the organising categories, while the content and boundaries of each code, and the textual cues taken to instantiate it, were continually checked against, and refined by, the data themselves. Where coverage did not map neatly onto the three archetypes, for instance, instances of delegitimation through scandal, ridicule, or reported illness that intersected with, but were not reducible to, the warrior frame, these were retained as distinct, emergent sub-themes (e.g., Political Delegitimation, Excessive Ambition, Scandalisation of Female Leaders) rather than forced into the original typology, and are reported as such in Table 1 and in the Findings.

**Table 1:** Coding frame for media analysis of Grace Mugabe, Nkosazana Dlamini-Zuma, and Joyce Banda.

Code	Description	Subcategories	Example articles/Citations
Dynastic Succession	Frames Grace Mugabe’s political role as part of a dynastic succession, emphasising her association with Robert Mugabe.	Wife-as-heir, Dynastic continuity	“Mugabe ‘anoints’ Grace” (NewsDay Zimbabwe, 2014); “Grace gets ‘endorsement’ for Presidency” (NewsDay Zimbabwe, 2014)
Heir Apparent	Portrayal of Grace Mugabe as the “heir apparent” to Robert Mugabe’s presidency, focusing on her role as successor.	Dynastic succession, Political inheritance	“Mugabe lines up Grace for Cabinet” (NewsDay Zimbabwe, 2014)
Political Legitimacy via Spousal Link	Frames Grace Mugabe’s legitimacy as derived from her marriage to Robert Mugabe rather than her own political merit.	Wife-as-heir, Political inheritance, Dynastic legitimacy	“Grace eyes Defence Ministry” (NewsDay Zimbabwe, 2014)
Caretaker/Transitional Leader	Frames Joyce Banda’s leadership as provisional or transitional, focusing on her role as a caretaker leader.	Visionary leader, Incomplete leadership, Interim leader	“Joyce Banda promises to elevate chiefs” (Malawi24, 2018); “Joyce Banda to launch 2019 presidential bid” (Malawi24, 2018)
Incomplete Visionary	Portrayal of Joyce Banda as a visionary leader, but one whose vision is framed as “unfinished” or “aspirational.”	Incomplete visionary, Unfinished business, Aspirational leader	“It is possible: Joyce Banda’s People’s Party Manifesto links Malawi2063 vision” (Malawi24, 2014); “Joyce Banda wants to complete development agenda” (Malawi24, 2018)
Patriarchal Subordination	Frames Joyce Banda’s leadership as linked to patriarchal tradition, such as elevating chiefs rather than transformative change.	Maternal leadership, Caretaker role, Patriarchal endorsement	“Joyce Banda promises to elevate chiefs” (Malawi24, 2018); “Joyce Banda to launch 2019 presidential bid” (Malawi24, 2018)
Liberation Mother	Frames Nkosazana Dlamini-Zuma as a continuation of ANC legacy and liberation struggle, positioning her as a maternal figure.	Liberation motherhood, ANC loyalist, Protective figure	“Dlamini-Zuma returns home after years in Addis Ababa at the AU” (Louw-Vaudran, 2017); “MKMVA, Dlamini-Zuma call for Ramaphosa to step down” (Siyamtanda Capa, 2025)
Liberation Legacy	Portrayal of Dlamini-Zuma’s leadership through her association with the ANC’s liberation legacy and traditional values.	Liberation legacy, ANC continuity, Patriarchal loyalty	“ISS Today: Dlamini-Zuma shuffles out of Addis—and into SA’s Cabinet” (Louw-Vaudran, 2017); “Dlamini-Zuma still in ANC presidency race” (Siyamtanda Capa, 2025)
Caretaker Role in ANC	Frames Dlamini-Zuma’s candidacy as primarily a caretaker position to uphold ANC traditions.	Caretaker leader, Party loyalty, Institutional absence	“What now for women seeking ANC top 6 positions?” (Siyamtanda Capa, 2025)
Political Delegitimation	Frames Grace Mugabe and Joyce Banda as morally or politically discredited through negative labeling or scandal.	Scandalisation, Corruption, Moral questioning	“Grace seriously ill—Mugabe” (NewsDay Zimbabwe, 2014); “Joyce Banda is a loser—DPP” (Malawi24, 2018); “Joyce Banda’s PP main beneficiary of Cashgate” (Malawi24, 2018)
Excessive Ambition	Frames Grace Mugabe’s political aspirations as excessive or unfit for the role she was seeking.	Excessive ambition, Unfit leadership, Warrior archetype	“I will spill blood—Grace” (NewsDay Zimbabwe, 2014); “Only a gun can stop me” (NewsDay Zimbabwe, 2014)
Scandalisation of Female Leaders	Frames Joyce Banda as a weak, corrupt leader by linking her to scandals and ridicule.	Corruption, Weakness, Ridicule	“Joyce Banda is ‘brainless’—PP supporters” (Malawi24, 2018); “Joyce Banda coming this October like a thief” (Malawi24, 2018)
Warrior Leadership	Frames Grace Mugabe as a warrior but in a delegitimizing way, emphasising her aggressiveness and political audacity.	Warrior frame, Excessive aggression, Militarised portrayal	“Grace leads 2017 noisemakers pack” (NewsDay Zimbabwe, 2014); “I will spill blood—Grace” (NewsDay Zimbabwe, 2014)
Patriarchal Marginalisation	Frames Nkosazana Dlamini-Zuma as secondary to male figures, either through comparisons or by situating her leadership in terms of male approval.	Patriarchal marginalisation, Masculinist standards, Secondary leader	“Ramaphosa lagging, Dlamini-Zuma edges forward in race to win” (Siyamtanda Capa, 2025); “What now for women seeking ANC top 6 positions?” (Siyamtanda Capa, 2025)
Patriarchal Moralization	Frames Joyce Banda’s leadership as unfit or morally questionable, using derogatory labels to undermine her authority.	Delegitimation, Moral questioning, Political failure	“Joyce Banda is a loser—DPP” (Malawi24, 2018); “Joyce Banda’s PP main beneficiary of Cashgate” (Malawi24, 2018)

The themes that emerged were grouped into broader categories reflecting patriarchal discourses and media framing techniques. These categories were then analysed for similarities and differences across the three countries, Zimbabwe, Malawi, and South Africa. This comparative analysis enabled the identification of cross-country patterns as well as country-specific nuances in the media’s portrayal of female political candidates (Tembo, 2024; Ncube and Yemurai, 2020).

A critical aspect of the analysis involved considering the political orientations of the news outlets. *NewsDay* (Zimbabwe) is known for its government-aligned stance, often framing political figures through the lens of national loyalty and legacy. In contrast, *IOL* (South Africa) reflect more diverse political viewpoints, with some publications reflecting more critical or oppositional stances towards political leadership. *Malawi24*, as a regional outlet, provides insight into local political dynamics, particularly in the context of Joyce Banda’s leadership. The varying political orientations of these outlets shaped the way each woman was framed, either as a political heir or as an exception to patriarchal norms.

Beyond editorial stance, the three outlets are embedded in markedly different national media ecosystems, which shapes how their coverage should be read comparatively. *NewsDay* operates within a Zimbabwean press environment historically constrained by restrictive media legislation and state pressure on privately owned titles, even though the outlet positions itself editorially as independent of the ruling party; its readership is concentrated among urban, English-literate, internet-connected audiences. *IOL* sits within South Africa’s comparatively pluralistic and commercially competitive press market, aggregating content from multiple regional and national titles with differing editorial lines, which helps explain the diversity of viewpoint noted above. *Malawi24* operates within a smaller and more recently established online news sector, in a national broadcasting and print landscape that remains heavily influenced by the state, and against a backdrop of comparatively limited internet penetration outside urban centres. These differences in ownership structure, regulatory context, and audience reach are treated here as part of the interpretive backdrop against which the findings should be read, rather than as variables held constant across cases, consistent with the qualitative, case-oriented design of this study.

The coding frame below (Table 1) was developed to guide the qualitative content analysis of media portrayals of Grace Mugabe, Nkosazana Dlamini-Zuma, and Joyce Banda in relation to their political identities, leadership aspirations, and public portrayals through historical and cultural archetypes, as well as patriarchal discourses.

Operationalising the codes in Table 1 required moving from broad thematic labels to specific, identifiable textual cues. For example, the code “Dynastic Succession” was built from articles whose headlines or lead paragraphs used kinship or inheritance vocabulary (e.g., “anoints,” “heir apparent,” “lines up”) to describe Grace Mugabe’s relationship to Robert Mugabe’s office, rather than describing her independent platform or policy positions; the presence of such vocabulary, rather than the topic of succession in the abstract, was the cue used to assign an article to this code. Similarly, “Caretaker/Transitional Leader” was built from recurring lexical markers of incompleteness and futurity (“promises,” “finish what she started,” “complete”) in coverage of Joyce Banda, while “Patriarchal Marginalisation” was built from comparative constructions that positioned Nkosazana Dlamini-Zuma’s progress relative to a male rival (e.g., “edges forward,” “lagging”) rather than describing her campaign in its own terms. This cue-level operationalisation is illustrated at greater length in the Results section, where extended excerpts from the coded articles are analysed.

Coding also surfaced sub-themes that were not anticipated by the original wife/widow/warrior typology, most notably the recurring use of bodily and health-related framing (as in coverage of Grace Mugabe’s illness) and explicitly criminalising metaphors (such as the comparison of Joyce Banda’s return to politics to a thief “coming”). These emergent codes, captured in Table 1 under headings such as Political Delegitimation, Excessive Ambition, and Scandalisation of Female Leaders, intersect with, rather than simply confirm, the warrior archetype, and are treated in the Discussion as evidence that the typology, while theoretically grounded, remained open to revision through sustained engagement with the data.

## 5 Results

This section presents an analysis of media coverage for Mugabe, Dlamini-Zuma, and Banda. Employing Entman’s (1993) four framing elements, problem definition, causal attribution, moral judgment, and treatment recommendation, the study identifies recurring themes and discourses shaping their media portrayals. The findings are organised thematically to align with the research questions.

### 5.1 Dynastic succession and the “wife-as-heir apparent”

Grace Mugabe’s political identity is systematically constructed through gendered and dynastic frames that privilege relational authority over individual political agency. Drawing on Framing Theory, such representations operate through selective emphasis, foregrounding her proximity to Robert Mugabe while backgrounding her independent political capacity (Robert Entman framing theory). In parallel, Feminist Theory reveals how these discursive patterns reproduce patriarchal archetypes, particularly the “wife/widow” trope, which positions women’s political legitimacy as derivative of male authority. Within this framework, Grace Mugabe is consistently framed not as an autonomous actor but as a conduit of dynastic continuity.

This construction is evident in the article “Mugabe ‘anoints’ Grace,” where it is stated: “PRESIDENT Robert Mugabe appears to have anointed his wife, Grace, as his possible successor after describing her as “fireworks” and ‘well-seasoned now’ to withstand the political heat within and outside the ruling Zanu PF party circles” (Ncube, 2015). The term “anointed” invokes religious and monarchical symbolism, suggesting a transfer of power grounded in personal endorsement rather than democratic legitimacy. The descriptors “fireworks” and “well-seasoned now” further situate her political readiness within a narrative of validation by her husband, reinforcing a frame in which her authority is conferred rather than earned.

A similar dynastic logic underpins “Grace gets ‘endorsement’ for Presidency,” which reports: “A Zanu PF faction linked to First Lady Grace Mugabe has “endorsed” her as President Robert Mugabe’s heir apparent, NewsDay has learnt. In the latest twist in the party’s succession war some party members have vowed to ensure her political rise.” (Matenga, 2015). The phrase “heir apparent” is a salient framing device that naturalises succession as hereditary and pre-determined, thereby constraining interpretations of her candidacy as independent or merit-based. Instead, her political trajectory is embedded within factional dynamics and elite orchestration, consistent with feminist critiques of women’s marginalisation within male-dominated political networks.

The narrative of passivity is further reinforced in “Mugabe lines up Grace for Cabinet,” where it is reported: “PRESIDENT ROBERT Mugabe is planning to appoint First Lady Grace Mugabe as the new Minister of Women Affairs after he did not name anyone to succeed Oppah Muchinguri who was yesterday sworn in as the new Minister of Higher and Tertiary Education” (Matenga and Mushava, 2014). The construction “lines up” implicitly removes her agency, depicting her as the object of political placement rather than an active participant. This linguistic framing aligns with feminist analyses of how women are grammatically positioned as recipients of action within political discourse.

Finally, in “Grace eyes Defence Ministry,” the claim that she was “prodding President Mugabe to appoint her Defence Minister” frames her ambition as informal and transgressive rather than legitimate. The term “prodding” diminishes the seriousness of her political aspiration, particularly within a male-dominated security portfolio, thereby reinforcing gendered boundaries around acceptable leadership domains.

Collectively, these verbatim representations consolidate a discursive regime in which Grace Mugabe is positioned as a “wife-as-heir apparent,” whose authority is constructed as derivative, mediated, and contingent upon spousal association. Such framing not only privileges dynastic continuity but also sustains patriarchal norms that constrain women’s political agency within postcolonial media landscapes.

### 5.2 Caretaker and the visionary but incompetent leader

Malawi24’s representation of Joyce Banda constructs a gendered political persona shaped by the intersecting frames of caretaker and visionary-yet-incomplete leader. From a framing theory perspective, the repetition of lexical cues such as “possible,” “promises,” “complete,” and “finish what she started” organises public interpretation around uncertainty, continuity, and provisional leadership rather than decisive executive authority. These are not neutral descriptors; they structure Banda’s leadership as emergent and conditional, encouraging audiences to read her political project as always in process, never fully realised. Feminist media theory deepens this interpretation by showing how women leaders are frequently inserted into symbolic roles associated with care, moral repair, and social uplift, while being denied the discursive markers of firmness, finality, and sovereign command typically granted to men.

This is visible in “It is possible: Joyce Banda’s People’s Party Manifesto links Malawi2063 vision,” where she is quoted as saying: “It is possible to transform Malawi,” distils the campaign brand into a hopeful imperative that converts critique into forward motion” (Munthali, 2025). Although this phrase appears optimistic, framing theory suggests that the wording “it is possible” encodes contingency rather than certainty. Her political imagination is therefore presented as aspirational and desirable, but not fully authoritative. Through a feminist lens, this becomes significant because women’s leadership is often narrated as hope-giving rather than power-exercising, thereby softening executive identity into inspirational symbolism.

A similar pattern appears in “Joyce Banda promises to elevate chiefs,” where the article states: “Let me remind you, my fellow Malawians, during the PP era, we elevated a good number of chiefs, including here in Dedza. We will continue with that next year,” she added…Former Malawi President Dr. Joyce Banda says she will elevate chiefs across the country if she returns to the presidency in 2019” (Malawi24 Reporter, 2018). Here, her leadership is framed through care, continuity, and deference to customary structures. The emphasis on “promises” and future return places her authority in a deferred register, while the focus on chiefs symbolically ties her leadership to custodianship rather than structural transformation.

This incompleteness is sharpened in “Joyce Banda wants to complete development agenda,” which reports that “She added that if elected, she will complete phase 2 of her development agenda, which she started implementing when she was president of Malawi from 2012 to 2014.” The same article also reported that, Former President Joyce Banda says she wants the presidency in 2019 to complete her development agenda that will see people in rural areas getting out of poverty (Kondowe, 2018). Likewise, “Joyce Banda to launch 2019 presidential bid” frames her return as an effort to “finish what she started.” Collectively, these framings deny closure, recasting Banda as permanently transitional: a nurturing reformer, but never a fully accomplished executive.

### 5.3 Liberation motherhood and dynastic continuity

Nkosazana Dlamini-Zuma’s (NDZ) leadership is consistently mediated through gendered and institutionalised frames that prioritise symbolic identity over political autonomy. Drawing on Framing Theory, these representations function as interpretive schemas that structure audience understanding by selecting and emphasising particular aspects of reality while marginalising others (Robert Entman’s framing theory). In parallel, Feminist Theory illuminates how such framing practices reproduce patriarchal norms by situating women leaders within restrictive archetypes, notably the trope of “liberation motherhood” and the notion of dynastic continuity. Within this discursive terrain, NDZ is positioned less as an autonomous political actor and more as a symbolic custodian of the African National Congress (ANC) legacy.

Media narratives from *IOL* between 2017 and 2024 demonstrate this pattern with notable consistency. In the article “‘I am standing and I am going to win,’ says Dlamini-Zuma in ANC presidential race” (2022), NDZ’s candidacy is framed through the label “presidential hopeful,” a term that subtly diminishes political authority and situates her within a lineage-bound trajectory rather than as a transformative contender. The emphasis on contestation within established party hierarchies reinforces what Entman (1993) identifies as frame selection: her ambition is acknowledged, yet contained within procedural and institutional continuity.

The trope of liberation motherhood is particularly salient in coverage involving military veterans. In “MK vets defend high-level security for Dlamini Zuma” (2017), NDZ is portrayed as a protected figure, “defended” and shielded, thus aligning her with a maternal guardian role over the liberation movement. Similarly, in “MK veterans in show of unity over ANC succession issue” (2017), debates over her leadership are embedded within narratives of succession and protection of struggle values. Feminist scholarship argues that such maternal framing constrains women’s leadership by associating authority with care, continuity, and moral stewardship rather than innovation or rupture (Coffie and Medie, 2021).

Dynastic framing is further reinforced in retrospective coverage. In “Dr Nkosazana Dlamini Zuma leaves behind a rich legacy” (2024), her departure from Parliament is narrated as a culmination of service to the ANC, emphasising loyalty and embeddedness within party structures. The phrase “rich legacy” situates her within a lineage of institutional continuity, effectively subsuming individual agency into collective history. Even in active political contexts, such as “Dlamini Zuma still in ANC presidency race” (2022), her expressed “optimism” is framed procedurally, reinforcing adherence to organisational rituals rather than articulating a distinct political vision.

These frames construct NDZ as both a maternal custodian and dynastic vessel, thereby limiting the discursive space for recognising her as an independent political strategist. This reflects broader gendered patterns in post-liberation media systems, where women leaders are symbolically elevated yet substantively constrained.

### 5.4 Delegitimation, pejoratives, and patriarchal moralization

Media representations of Grace Mugabe reveal a systematic pattern of delegitimation structured through gendered framing devices that trivialise, personalise, and morally regulate her political identity. Through Framing Theory, these portrayals can be understood as strategic selections that foreground particular attributes, disruption, fragility, and excess, while obscuring political substance. Simultaneously, Feminist Theory exposes how such discourses reproduce patriarchal logics that discipline women in power by casting them as deviant from normative expectations of femininity. Importantly, these framings also intersect with the cultural archetype of women as “warriors,” but this identity is rearticulated in a delegitimising register, where assertiveness is recoded as aggression and threat.

This is evident in “Grace leads 2017 noisemakers pack,” where it is stated: “FORMER First Lady Grace Mugabe leads the pack of discredited politicians who make up the list of 2017’s noisemakers after an inglorious end to a short-lived, but eventful political career.” The label “noisemakers” functions as a powerful framing cue that trivialises political speech, reducing her voice to disruptive excess rather than legitimate engagement. From a feminist perspective, this reflects a long-standing patriarchal trope in which outspoken women are delegitimised as irrational or loud, thereby disciplining their presence in the public sphere.

Delegitimation is further reinforced through bodily framing in “Grace seriously ill, Mugabe,” where it is reported that “PRESIDENT ROBERT Mugabe yesterday touched down at Harare International Airport from his month-long holiday in the Far East, minus his wife, First Lady Grace, whom he said remained behind as she was suffering from appendicitis.” The article continues: “Although Mugabe seemed to be downplaying his wife’s illness, medical experts who spoke on condition of anonymity yesterday alluded that she very likely had cancer of the colon.” Here, the foregrounding of illness constructs a narrative of fragility and dependence, aligning with feminist critiques of how women’s bodies are politicised to undermine authority. Unlike male counterparts, whose health is seldom centralised in political evaluation, Grace’s credibility is tethered to perceived physical weakness.

The personalisation of blame is evident in “Grace derails EU sanctions removal,” which asserts that “the European Union’s decision to retain the travel embargo on Zimbabwe stemmed from Grace’s recent land grabs at Manzou Farm.” This framing isolates her as the causal agent of complex geopolitical outcomes, reinforcing gendered narratives of women as destabilising forces within political systems.

However, the most striking dimension emerges in militarised framings. In “I will spill blood—Grace,” she is quoted as saying: “Nobody will remove me from the farm which I took. Blood will be spilt if anyone attempts to remove me from that farm. I took that farm personally because even after I told ministers and government officials that I wanted a farm, they did not allocate me land and instead told me they thought I was joking.” Similarly, in “Only a gun can stop me,” the report states that “FIRST Lady Grace Mugabe yesterday warned hawks in the faction-ridden Zanu PF party she would only be silenced by a gun and would stop at nothing in admonishing divisive behaviour by party members though she did not harbour any presidential ambitions.” While these statements invoke the cultural archetype of the woman as a “warrior,” a figure of resistance, strength, and defiance, framing theory reveals that this identity is selectively encoded as excessive and pathological. Rather than legitimising her assertiveness, the media rearticulates the warrior trope as dangerous transgression, contrasting sharply with how similar rhetoric by male politicians is often normalised as strength.

Collectively, these framings reproduce patriarchal benchmarks of leadership, where women’s political expression is either trivialised, pathologised, or rendered morally suspect. The warrior archetype, instead of empowering, is reframed to discipline, thereby constraining the legitimacy of women’s authority in political life.

### 5.5 Marginalisation through patriarchal benchmark

Media constructions of Nkosazana Dlamini-Zuma (NDZ) reveal a layered gendered framing in which her political identity is negotiated through the intersecting tropes of the “wife,” the “widow,” and the “warrior,” yet consistently subordinated within patriarchal benchmarks of leadership. From the perspective of Framing Theory, these portrayals operate through relational and comparative cues that define her candidacy in reference to male counterparts, thereby structuring meaning around dependency and insufficiency (Robert Entman framing theory). Feminist Theory further clarifies that such discourses reproduce entrenched gender hierarchies, where women are required to negotiate legitimacy through roles historically coded as feminine, supportive, exceptional, or morally symbolic, rather than authoritative and sovereign.

In “Ramaphosa lagging, Dlamini-Zuma edges forward in race to win,” NDZ is described as “edging forward,” a phrase that situates her progress within a comparative and subordinate frame. Rather than recognising independent political momentum, her candidacy is narratively tethered to Cyril Ramaphosa, reinforcing a “wife/widow” trope in which women’s political identities are relationally defined through proximity to dominant male figures. Even where movement is acknowledged, it is incremental and derivative, reflecting what feminist scholars identify as the discursive minimisation of women’s political agency.

This marginalisation is intensified in “Political analyst says Ramaphosa faction had been waiting for a chance to deal with Dlamini-Zuma” (Mafisa, 2025), where her role is constructed within factional contestation. Here, NDZ is not framed as a decisive “warrior” in political struggle, but rather as an object within strategic manoeuvring, someone to be “dealt with.” The warrior archetype, historically associated with resistance and strength, is thus reconfigured in a diminished register, where her participation in political contestation is stripped of agency and recast as vulnerability within male-dominated power games.

Gendered exceptionalism is further reproduced in “What now for women seeking ANC top 6 positions?” (Capa, 2022), where the very framing of the question positions women’s leadership as anomalous. By asking whether women like NDZ can access top leadership, the article reinforces a “widow” trope of waiting, legitimacy-through-absence, or conditional inclusion, rather than normalising women’s authority. This reflects feminist critiques that women in politics are constructed as perpetual outsiders who must continually justify their presence within masculinised institutions.

In “Nkosazana Dlamini-Zuma fears ‘auctioning of ANC’,” her critique is quoted as: “My sense is that money is still playing a big role. There are people who want to auction, and I think the committee must do a lot more.” She added that there were financial spreadsheets being “thrown around” which indicated something was amiss. While this statement engages substantively with governance concerns, framing theory reveals how its presentation recodes her intervention as defensive. Rather than amplifying her as a “warrior” confronting corruption, the discourse subtly aligns her critique with political vulnerability, thereby weakening its legitimacy.

These framings produce a constrained discursive field in which NDZ oscillates between the roles of wife (relational and secondary), widow (exceptional and conditional), and warrior (assertive yet delegitimised). The warrior archetype, rather than empowering, is selectively reframed to appear reactive or excessive, while patriarchal benchmarks of leadership remain firmly masculinised. Consequently, her political presence is rendered secondary, contingent, and continually measured against standards that structurally privilege male authority.

### 5.6 Delegitimation and scandalisation through patriarchal discourses

Media portrayals of Joyce Banda demonstrate a patterned delegitimation structured through gendered frames that combine ridicule, scandalisation, and moral judgement. From the perspective of Framing Theory, repeated lexical cues such as “loser,” “brainless,” and “Cashgate” function as organising devices that anchor audience interpretation around failure, incompetence, and corruption, irrespective of evidentiary complexity (Robert Entman framing theory). Feminist Theory further reveals how these framings reproduce patriarchal standards that subject women leaders to intensified moral scrutiny and personalisation, often through the intersecting tropes of the “wife,” the “widow,” and the “warrior.” In Banda’s case, these tropes are mobilised to diminish authority: the widow as inheritor rather than originator, and the warrior recast as deviant or excessive.

In “Joyce Banda is a loser—DPP,” her political identity is reduced to the label “loser.” This framing collapses her leadership into electoral defeat, erasing policy substance and reinforcing a discourse of disposability. Similarly, “Joyce Banda is ‘brainless’—PP supporters” amplifies the insult “brainless,” a deeply gendered term that delegitimises intellectual capacity. Feminist analysis suggests that such descriptors are not incidental but are part of a broader symbolic economy in which women’s cognition and rationality are routinely questioned, positioning them as unfit for leadership.

The “Cashgate” scandal operates as a dominant interpretive frame. In “Joyce Banda’s PP main beneficiary of Cashgate—convict,” the assertion that “Banda’s People’s Party benefited the most from the looted funds” constructs associative guilt. Framing theory indicates that the repetition of this linkage transforms the allegation into a perceived fact, embedding corruption as a defining attribute of her political identity. Notably, this scandalisation exceeds individual accountability and becomes a shorthand for evaluating her presidency, overshadowing governance achievements.

Criminalisation is further evident in “Joyce Banda coming this October like a thief,” where the metaphor “like a thief” frames her political return as illicit and threatening. This imagery resonates with patriarchal anxieties surrounding women who re-enter or reclaim power, particularly those who disrupt established hierarchies. Rather than being framed as a resilient “warrior” returning to contest political space, her reappearance is constructed as suspect and destabilising.

Across these examples, the warrior archetype, typically associated with resilience and resistance, is inverted. Banda’s attempts to reassert political presence are not framed as strength but as transgression, while her widow-associated succession is used to question legitimacy. Collectively, these framings entrench a discursive regime in which her leadership is trivialised, moralised, and scandalised, thereby constraining democratic recognition of her as a credible political actor.

While Grace Mugabe, Joyce Banda, and Nkosazana Dlamini-Zuma each gained prominence in different political settings, there are notable similarities and differences in how their media portrayals were influenced by gendered expectations (Mangena, 2022; Muringa and McCracken, 2021; Kondowe et al., 2014). All three women were viewed through the lens of their connections to influential male figures: Grace Mugabe as the wife of Robert Mugabe, Joyce Banda as the political successor to her late husband, and Nkosazana Dlamini-Zuma as the ex-wife of Jacob Zuma (Mangena, 2022; Muringa, 2026; Kondowe et al., 2014). These familial ties often overshadowed their individual political accomplishments, with their legitimacy questioned based on their male counterparts’ influence rather than their own political skills (Mangena, 2022; Muringa and McCracken, 2021). However, the key difference lies in their political trajectories. While Banda’s presidency in Malawi was constitutionally mandated following her predecessor’s death, Dlamini-Zuma’s candidacy for ANC president was a voluntary decision and marked a more direct challenge to the male-dominated leadership of South Africa’s ruling party (Muringa and McCracken, 2021; Kondowe et al., 2014). Grace Mugabe’s leadership ambitions were rooted in dynastic politics and the broader context of authoritarian rule in Zimbabwe, which set her apart from both Banda and Dlamini-Zuma in terms of political legitimacy and public reception (Mangena, 2022). These differences highlight how the media framing of women politicians is influenced not only by gendered archetypes but also by the specific political and cultural contexts in which they operate (Mangena et al., 2021; Muringa and McCracken, 2021).

## 6 Discussion

This study reveals how the media portray Grace Mugabe, Nkosazana Dlamini-Zuma, and Joyce Banda within deeply entrenched patriarchal discourses that shape their political identities through gendered archetypes. Specifically, the study found that these women are consistently framed as “wives,” “widows,” or “warriors,” limiting their recognition as independent political leaders. This finding aligns with existing literature, which highlights the impact of patriarchal structures on women’s political agency (Msweli, 2023; Coffie and Medie, 2021). The use of gendered frames in media not only diminishes the legitimacy of these women as political leaders but also confines their political identities to predefined traditional roles rooted in historical and cultural gender norms. These results suggest that the media in Southern Africa continues to reinforce gendered hierarchies, portraying women as extensions of male authority or as exceptional figures who challenge the political status quo, but rarely as normative political leaders. From a Critical Language Awareness (CLA) perspective, these representations demonstrate that media language does not merely describe women’s political participation. It actively constructs the boundaries within which their authority, competence, and legitimacy can be understood. The recurring archetypes therefore illustrate the ideological and constitutive power of language identified by Darics et al. (2025), because they reproduce patriarchal assumptions while presenting them as ordinary descriptions of political reality.

The study found that Grace Mugabe’s political legitimacy was primarily framed as an extension of her husband Robert Mugabe’s rule. This framing aligns with the work of Sreberny and van Zoonen (2000), who noted that women in politics are often depicted through their familial connections, positioning them as extensions of male authority rather than independent figures. The media’s use of terms such as “Mugabe ‘anoints’ Grace” and “Grace gets ‘endorsement’ for Presidency” clearly positions her as the heir to her husband’s political legacy, diminishing her agency and reinforcing patriarchal norms that limit women’s autonomy in the political sphere. Such lexical choices are not politically neutral. The verb “anoints,” for example, assigns political authority to Robert Mugabe and constructs Grace Mugabe as a passive recipient of power rather than an actor who possesses an independent political programme. This framing of Grace as a “wife-as-heir” highlights the persistent power dynamics in post-colonial African politics, where women’s roles are frequently reduced to symbols of continuity within patriarchal structures. These findings resemble studies in other post-colonial contexts, such as South Asia, where political wives are often viewed as successors to male-dominated leadership (Mangena, 2022). However, comparisons with other cases, such as those in Latin America, reveal notable differences, where female political leaders, although still framed within familial contexts, sometimes gain more autonomy (Agu, 2024).

The implications of this finding are significant. The media’s portrayal of Grace Mugabe within a dynastic and passive role perpetuates the idea that women in politics are legitimate only when linked to male political legacies. This reinforces the barriers women face in establishing themselves as independent political figures. The study suggests that, to break free from these patriarchal constraints, the media must challenge traditional gender roles and enable women to define their own political identities, as argued by Muringa and McCracken (2021) and Coffie and Medie (2021). Within CLA, such a shift would move media practice beyond recognising discriminatory language towards advocacy. It would require journalists and editors to name the inequality embedded in dynastic frames and deliberately adopt alternative descriptions that foreground women’s qualifications, policy positions, political records, and independent sources of legitimacy.

Similarly, Nkosazana Dlamini-Zuma’s media representation was influenced by the “warrior” archetype, but the study found that this did not empower her as expected. Instead of being portrayed as an independent leader, her candidacy was shown as secondary to Cyril Ramaphosa’s, with her progress described in relation to his. This reflects a broader trend identified in previous research, where women politicians are frequently portrayed relative to male counterparts rather than recognised for their own political ambitions (Muringa and McCracken, 2021). While Dlamini-Zuma was positioned as a “warrior” challenging the male-dominated ANC leadership, the media also framed her authority as anchored in ANC loyalty, emphasising her role as a “liberation mother” rather than as an independent political force. Although the warrior frame appears positive, it still marks female leadership as exceptional and conflict-based. Male leadership remains the implicit norm against which her political identity is evaluated.

This finding aligns with Coffie and Medie’s (2021) argument that women leaders in post-colonial African politics are often depicted as moral custodians of their political movements rather than as proactive political agents. The implications are important for understanding the limitations placed on women leaders within political organisations. The study indicates that women’s leadership, even when represented as challenging male dominance, is often restricted by the expectation that women must conform to established political structures and movement identities. This echoes Msweli’s (2023) observation that women leaders are frequently viewed as symbolic figures within their political movements, with their authority deriving from their movement roles rather than from independent political visions. These insights add to the literature on the “double bind” faced by women in politics, as discussed by Krook (2020), where women are expected either to follow male leadership or to demonstrate exceptional qualities, but are rarely permitted to occupy the same normative political space as men. A CLA-informed alternative would therefore avoid measuring Dlamini-Zuma’s political viability primarily against Ramaphosa or reducing her leadership to maternal and liberation-based symbolism. Advocacy would involve reframing her as a political actor whose competence can be evaluated through her policy agenda, administrative record, leadership approach, and proposed national programme.

The media framing of Joyce Banda also reflects a pattern of marginalisation. She was depicted as a “caretaker” and “visionary,” but her leadership was frequently portrayed as “unfinished” or “aspirational.” The study found that phrases such as “unfinished business” and “It is possible” reduced her political authority, casting her as a transitional figure rather than a decisive leader. This framing aligns with Ross’s (2017) account of the double bind, in which women leaders are praised for symbolic qualities such as nurturing or caregiving, while their authority is undermined when they fail to conform to patriarchal expectations of decisiveness. Banda’s leadership was therefore framed as requiring continuing external validation, which echoes the work of Coffie and Medie (2021), who argue that women leaders are often placed in positions where they must continually prove their competence.

The implications of this finding suggest that women in politics are rarely permitted to assert their authority fully without being framed in ways that conform to patriarchal expectations. The depiction of Banda as a “caretaker” and “visionary” reflects a broader pattern in which women leaders are expected to be nurturing and transitional figures rather than decisive leaders capable of implementing long-term change. This is consistent with previous research on women politicians in other regions, including Latin America, where female leaders are often framed as caregivers or moral authorities (Msweli, 2023). However, unlike more empowering portrayals in some Western contexts, where female leaders may be permitted to assert authority within a more normative political space, Banda’s portrayal remained constrained by gendered expectations. CLA draws attention to the consequences of these seemingly positive labels. Terms such as “caretaker” and “visionary” can symbolically praise a woman while simultaneously withholding recognition of her material achievements and executive authority. An advocative shift would replace these limiting labels with reporting that assesses Banda according to the same standards of policy delivery, political strategy, institutional leadership, and accountability applied to male presidential candidates.

Media portrayals of Grace Mugabe, Nkosazana Dlamini-Zuma, and Joyce Banda therefore reflect a broader Southern African tendency to frame women politicians through restrictive gendered archetypes. Existing literature shows that women are commonly positioned relative to men or presented as political exceptions rather than normative leaders (Krook, 2020; Coffie and Medie, 2021). Muringa and McCracken (2021) similarly observe that media reporting frequently marginalises women politicians and restricts their agency. Comparatively, Latin American leaders such as Cristina Fernández de Kirchner and Dilma Rousseff are also subject to gendered stereotypes, although their media portrayals sometimes receive greater recognition of their direct political authority due to different cultural and institutional contexts (Agu, 2024). Conversely, in the Middle East and North Africa, women politicians are frequently depicted as wives or daughters of male leaders, further constraining their political agency (Omar, 2025). These findings demonstrate that entrenched gender norms continue to shape media portrayals across regions.

The contribution of CLA is particularly important at this point because awareness of discriminatory framing should not be treated as the final outcome of analysis. Darics et al. (2025) conceptualise CLA as a progression from awareness to agency, advocacy, and activism. Within this framework, advocacy involves speaking up, naming injustice, framing issues clearly, sharing knowledge beyond academic audiences, and using discourse deliberately to promote equity. It entails taking an ethical position and a willingness to intervene in harmful language practices rather than merely documenting them.

A shift in the media portrayal of female presidential candidates would constitute advocacy in this CLA sense. Such advocacy would not require journalists to support candidates or abandon professional scrutiny. Instead, it would require them to recognise that reporting always involves selection, framing, and positioning. Ethical reporting would make these choices reflexive and accountable. It would avoid defining women principally through marital status, male relationships, motherhood, widowhood, or exceptional struggle. It would instead foreground political experience, policy preferences, leadership records, ideological positions, institutional competence, and public accountability. As Darics et al. (2025) emphasise, advocacy is not synonymous with partisanship. It reflects a responsibility to challenge harmful norms, amplify marginalised voices, expose ideological assumptions, and offer more equitable alternative discourses.

The proposed gender-sensitive reporting framework therefore represents a practical form of CLA advocacy. It translates critical recognition of gendered language into professional intervention. By encouraging journalists and editors to identify restrictive archetypes, question whose interests those frames serve, and reconstruct political narratives around substantive leadership, the framework can contribute to more equitable representation. This movement from identifying patriarchal discourse to reforming media practice illustrates CLA as a future-oriented competence. It equips media professionals not only to “see” the ideological work performed by language, but also to use language more ethically to reshape the political realities available to women. Greater media accountability and more nuanced portrayals could consequently help dismantle the discursive barriers that continue to restrict women’s political participation and recognition as legitimate presidential contenders.

## 7 Conclusion

This study examined how Southern African media represent women presidential candidates through gendered archetypes that restrict their political agency. The findings show that Grace Mugabe, Joyce Banda, and Nkosazana Dlamini-Zuma were framed as the “wife,” “widow,” or “warrior.” These portrayals emphasised familial relationships, political dynasties, and exceptionalism rather than recognising women as legitimate and autonomous political leaders. Consequently, media framing reproduces patriarchal assumptions and influences how women’s competence, authority, and suitability for leadership are evaluated. From a Critical Language Awareness (CLA) perspective, these findings demonstrate that language does not merely report political reality; it actively constructs it. CLA enables journalists, editors, and audiences to identify the lexical choices, frames, and ideological assumptions through which gender inequality is normalised. However, awareness must lead to advocacy through deliberate changes in reporting practices (Darics et al., 2025). Media coverage should assess women by their political experience, policy positions, leadership records, institutional competence, and accountability, rather than by their marital status or relationships with men. The proposed CLA-informed, gender-sensitive reporting framework therefore translates critical awareness into practical advocacy. Future research should examine whether such interventions influence journalistic practices, voter perceptions, and the political aspirations of young women across Southern Africa.

### 7.1 Implication for theory and practice

The findings of this study have significant implications for both theory and practice. Theoretically, the study contributes to feminist media theory and framing theory by highlighting how gendered archetypes- wife, widow, and warrior- continue to shape media portrayals of female political leaders in Southern Africa. By applying Entman’s (1993) framing elements, the research demonstrates how media framing not only restricts women’s political agency but also perpetuates patriarchal discourses that limit their legitimacy as independent leaders. This challenges the conventional understanding of media representation as neutral, showing instead that media narratives are deeply intertwined with historical and cultural gendered norms. In practice, the study calls for greater media accountability and a shift towards more diverse and equitable representation of women in political leadership. For journalists and media practitioners, this research underscores the need to be conscious of the implicit biases that shape coverage of female politicians and to prioritise narratives that recognise women’s leadership as autonomous and valid. By doing so, the media can play a crucial role in advancing gender equality in political representation, enabling more women to be seen and accepted as legitimate leaders in Southern Africa and beyond.

### 7.2 Limitation of the study

A key limitation of this study is its dependence on a limited number of online news outlets from Southern Africa, which might not fully reflect the diversity of media narratives across the region or consider variations in local contexts. Furthermore, the study concentrates exclusively on the media portrayal of female presidential candidates, potentially neglecting other forms of political participation and the subtle media depictions of women in lower political offices. It also primarily investigates English-language sources, risking the exclusion of representations of women in non-English media outlets. Relatedly, the corpus of 122 articles is modest and unevenly distributed across the three outlets, and was analysed using an interpretive, case-oriented logic rather than a frequency-based content-analytic one; the findings should therefore be read as illustrative of recurring discursive patterns within each case rather than as statistically representative of media coverage in Malawi, Zimbabwe, or South Africa more broadly. Because the three leaders were covered by different outlets, in different decades, and within different national media and political systems, cross-country comparisons in this study are necessarily comparisons of archetypal patterns rather than of directly equivalent, time-matched samples; readers should be cautious about generalising findings from one country’s media system to another. Lastly, although ChatGPT was used as a tool to help identify themes, employing AI in qualitative analysis raises concerns about the software’s accuracy and understanding of context, which could influence the richness and depth of the final analysis.

## Data Availability

The original contributions presented in the study are included in the article/supplementary material, further inquiries can be directed to the corresponding author.
